# Modeling and Prediction of Thermal Deformation Errors in Fiber Optic Gyroscopes Based on the TD-Model

**DOI:** 10.3390/s23239450

**Published:** 2023-11-27

**Authors:** Jintao Xu, Ailing Tian, Hui Liu, Ying Liu

**Affiliations:** 1 Key Laboratory of Thin Film Technology and Optical Testing in Shaanxi Province, School of Opto-Electronic Engineering, Xi’an Technological University, Xi’an 710021, China; xujintao@opt.ac.cn; 2 Xi’an Zhongke Huaxin Measurement & Control Co., Ltd., Xi’an 710119, China; 3 School of Automation, Xi’an University of Posts & Telecommunications, Xi’an 710121, China; liuhui@xupt.edu.cn (H.L.); ly676@xupt.edu.cn (Y.L.)

**Keywords:** fiber optic gyroscope, thermal errors, prediction model, overfitting, biased regression

## Abstract

For a fiber optic gyroscope, thermal deformation of the fiber coil can introduce additional thermal-induced phase errors, commonly referred to as thermal errors. Implementing effective thermal error compensation techniques is crucial to addressing this issue. These techniques operate based on the real-time sensing of thermal errors and subsequent correction within the output signal. Given the challenge of directly isolating thermal errors from the gyroscope’s output signal, predicting thermal errors based on temperature becomes necessary. To establish a mathematical model correlating the temperature and thermal errors, this study measured synchronized data of phase errors and angular velocity for the fiber coil under various temperature conditions, aiming to model it using data-driven methods. However, due to the difficulty of conducting tests and the limited number of data samples, direct engagement in data-driven modeling poses a risk of severe overfitting. To overcome this challenge, we propose a modeling algorithm that effectively integrates theoretical models with data, referred to as the TD-model in this paper. Initially, a theoretical analysis of the phase errors caused by thermal deformation of the fiber coil is performed. Subsequently, critical parameters, such as the thermal expansion coefficient, are determined, leading to the establishment of a theoretical model. Finally, the theoretical analysis model is incorporated as a regularization term and combined with the test data to jointly participate in the regression of model coefficients. Through experimental comparative analysis, it is shown that, relative to ordinary regression models, the TD-model effectively mitigates overfitting caused by the limited number of samples, resulting in a substantial 58% improvement in predictive accuracy.

## 1. Introduction

The fiber optic gyroscope, based on the Sagnac effect, represents a new generation of high-performance inertial sensors. It has gained prominence as the preferred inertial instrument for medium- and high-precision inertial navigation systems due to its outstanding advantages, such as high accuracy, solid-state design, and high reliability. It finds extensive applications in various domains, including marine, terrestrial, aerial, and space sectors [1,2]. Currently, there are two primary technical approaches for fiber optic gyroscopes: the traditional fiber optic gyroscope with discrete packaging of core optical components and the integrated optical fiber gyroscope. With the rapid development of optoelectronics, microelectronics, and micro/nano-processing technologies, integrated optical chips that combine various functions such as emission, coupling, modulation, and detection have made significant breakthroughs. These integrated optical chips are widely used in the field of optical communications, especially silicon-based photonic devices that are compatible with traditional CMOS (Complementary Metal-Oxide-Semiconductor) processes. They offer advantages like compact size, high performance, and cost-effectiveness, making them a key technological solution for future photonic integration [3,4]. Whether it is the traditional discrete fiber optic gyroscope or the integrated optical fiber gyroscope, the optical fiber coil serves as the sensitive core component, and its performance directly impacts the engineering precision of fiber optic gyroscopes [5].

The temperature performance of the optical fiber coil has the most significant impact on the zero-bias error of the fiber optic gyroscope [6]. When the fiber optic gyroscope is used in high-temperature environments, temperature effects can cause geometric deformation of the optical fiber coil, deviating from its original output characteristics, resulting in thermal errors. For sensing devices whose working principles depend on geometric characteristics, thermal errors are an unavoidable source of error [7,8]. Currently, thermal error compensation is an effective means to reduce thermal deformation errors. It calculates the thermal error magnitude at various temperatures through algorithms and provides feedback compensation. Its advantage lies in not requiring hardware modifications; it can reduce thermal errors through software alone [9]. The key to thermal error compensation is the ability to obtain accurate thermal error values. However, for fiber optic gyroscopes, thermal errors are difficult to directly separate from the measured values. Therefore, it is feasible to indirectly predict thermal errors based on temperature. However, this approach requires the establishment of a mathematical model between thermal errors and temperature for the fiber optic gyroscope. In theory, if the thermal expansion coefficient of the optical fiber coil is known, it is possible to derive the additional phase error caused by temperature based on its working principle. However, the material composition of fiber optic gyroscopes is complex, including not only the core but also cladding, stress zones, inner coatings, and outer layers [10]. This complexity makes it difficult to determine the comprehensive thermal expansion coefficient when various materials are combined. Additionally, due to differences in manufacturing processes for different fiber optic gyroscopes and variations between production batches, it is challenging to establish a quantitative thermal error model for fiber optic gyroscopes purely through theoretical derivation.

In addition to pure theoretical derivation, data-driven methods are also an effective approach for modeling complex objects. For thermal deformation errors in fiber optic gyroscopes, it is possible to directly measure phase errors at different temperatures and model them based on data. This method has been widely applied in the study of thermal errors in numerical control machine tools [11], with the common practice being the establishment of a model between key points’ temperatures on the machine tool and thermal errors. Multiple regression benefits from its simplicity and fast computation speed, making it the most commonly used method. Mayr [12] and Zimmermann [13,14] utilized multiple regression algorithms to propose a thermal error compensation mechanism that updates the model online. It assesses the model’s effectiveness through periodic accuracy checks, supplements new measurement data if the model becomes ineffective, and rebuilds the model. Wei [15], using the Gaussian process regression algorithm, established an adaptive thermal error model that adjusts according to environmental changes. Machine tool structures vary widely, and some exhibit complex thermal deformation error patterns. Therefore, neural networks have also found extensive application. Li [16] combined stochastic theory, genetic algorithms, and the radial basis function neural network (RBFNN) algorithm to create a comprehensive model that considers thermal errors, temperature, and random disturbance factors, thereby improving the robustness of thermal error prediction. Liu [17,18] leveraged the long-term memory properties of LSTM neural networks to establish thermal error models, allowing multiple time data points to contribute to thermal error prediction and enhancing prediction accuracy. Additionally, some researchers [19] have based their work on convolutional neural networks, using deep learning to directly establish mathematical models between the overall machine tool temperature field and thermal errors.

In the aforementioned research on modeling thermal errors in numerical control machine tools, despite using different algorithms, they all employed the same fundamental strategy: increasing the number of modeling samples to enhance the model’s generalization properties and reduce overfitting. Overfitting [20] can lead the model to fit irrelevant information from the training data, such as errors, leading to a significant deterioration in the model’s predictive performance on data not included in the modeling process. The scarcity of modeling data samples is one of the reasons for the overfitting problem, and it is also a central challenge in modeling thermal deformation errors in fiber optic gyroscopes. Modeling thermal deformation errors in fiber optic gyroscopes is challenging because the measurement of these errors is difficult. Each temperature point measurement requires a relatively long wait time to allow the temperature field to reach equilibrium. For mass-produced products, it is challenging to perform intensive temperature interval sampling. Additionally, the performance of sensors can be highly unstable under frequent temperature fluctuations, necessitating frequent compensation and correction of the sensor’s own thermal errors. All these factors lead to data-driven modeling of thermal deformation errors in fiber optic gyroscopes, and it is necessary to overcome the overfitting problem caused by a small sample size.

In response to these challenges, this paper introduces a modeling algorithm that combines theoretical models with modeling data. Taking into account the stability of the fiber optic gyroscope’s material, it is possible to deduce the comprehensive thermal expansion coefficient of the optical fiber material and establish a theoretical model for thermal deformation errors in the fiber optic gyroscope through a single intensive temperature experiment. Subsequently, for different batches of fiber optic gyroscopes, while there may be process variations, the resulting characteristic changes can be distributed around the theoretical model following a certain probability density function. If a confidence probability is set, and it is believed that the occurrence of characteristics outside the confidence probability is a rare event, then the distribution of characteristic variations remains within a limited range around the theoretical model. This indicates that the theoretical model still contains valuable information. Consequently, when modeling, the theoretical model can be used as a regularization term, combined with small-sample data to address overfitting issues. Based on this, the algorithm proposed in this paper is referred to as the TD-model modeling algorithm, and its effectiveness is validated through experiments. Based on this approach, the algorithm proposed in this paper is referred to as the TD-model modeling algorithm, and its effectiveness has been experimentally validated.

## 2. The Principle of a Fiber Optic Gyroscope

**Figure 1:**
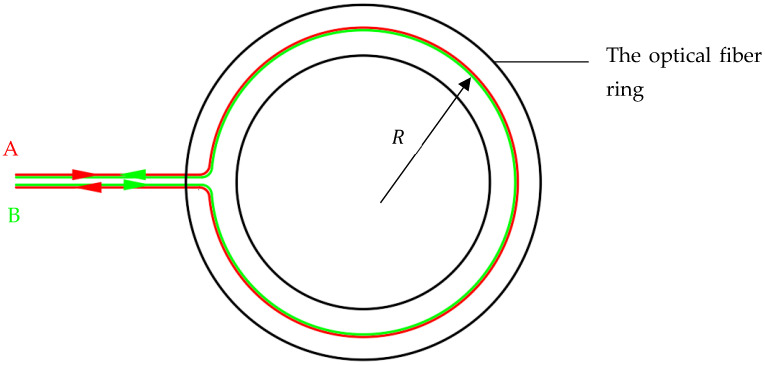
Fiber optic coil (Stationary state).

Figure 1 shows two beams of light, A and B, propagating along a fiber optic ring in different directions. In this setup, fiber optic A travels through the fiber optic ring in a clockwise direction, while fiber optic B travels in a counterclockwise direction. Both fiber optic beams follow a path with a radius of R within the fiber optic ring.

The geometric distance traveled by two optical fibers within a stationary optical fiber ring is equal, as follows.

(1) $${L_{A}}^{0}={L_{B}}^{0}=2\pi R$$

**Figure 2:**
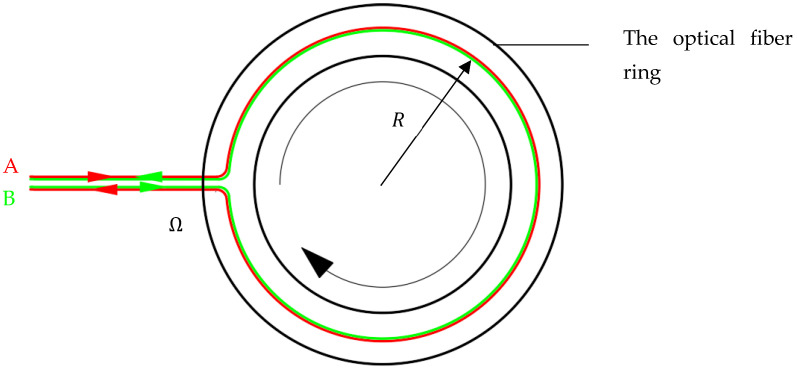
Optical fiber ring (Rotational state).

Now, the optical fiber ring rotates at a certain angular velocity Ω, such as clockwise, as shown in Figure 2.

According to the Sagnac effect, the geometric distance traveled by light beams A and B around the optical fiber ring is no longer equal, as follows.

(2) $$\left\{\begin{array}{c} L_{A}=2\pi R+R\Omega t_{A}\\ L_{B}=2\pi R-R\Omega t_{B} \end{array}\right.$$

In which, $L_{A}$ and $L_{B}$ represent the geometric distances traveled by light beams A and B around the optical fiber ring for one complete rotation, and $t_{A}$ and $t_{B}$ denote the time spent.

Therefore, when the optical fiber ring starts to rotate, there is a certain optical path difference between the two light beams at the exit position, resulting in a phase difference ∆Φ. By measuring the phase difference ∆Φ between the two light beams, the rotational angular velocity Ω of the optical fiber ring can be calculated.

According to the Lorentz–Einstein velocity transformation formula, the propagation velocities of light beams A and B in the rotating optical fiber ring no longer have a simple proportionality relationship with the refractive index. Specifically, it is as follows.

(3) $$\left\{\begin{array}{c} c_{A}=\frac{c_{0}/n+R\Omega }{1+R\Omega /\left(nc_{0}\right)}\\ c_{B}=\frac{c_{0}/n-R\Omega }{1-R\Omega /\left(nc_{0}\right)} \end{array}\right.$$

where $c_{A}$ and $c_{B}$ are the propagation velocities of light beams A and B within the optical fiber ring, $c_{0}$ is the speed of light in a vacuum, and *n* is the refractive index of the optical fiber ring.

Combining Equations (2) and (3), it can be seen that

(4) $$\left\{\begin{array}{c} t_{A}=\frac{L_{A}}{c_{A}}=\frac{\left(2\pi R+R\Omega t_{A}\right)\left(1+R\Omega /\left(nc_{0}\right)\right)}{c_{0}/n+R\Omega }\\ t_{B}=\frac{L_{B}}{c_{B}}=\frac{\left(2\pi R-R\Omega t_{A}\right)\left(1-R\Omega /\left(nc_{0}\right)\right)}{c_{0}/n-R\Omega } \end{array}\right.$$

(5) $$⟹\left\{\begin{array}{c} t_{A}=\frac{2\pi R\left(nc_{0}+R\Omega \right)}{{c_{0}}^{2}-{\left(R\Omega \right)}^{2}}\\ t_{B}=\frac{2\pi R\left(nc_{0}-R\Omega \right)}{{c_{0}}^{2}-{\left(R\Omega \right)}^{2}} \end{array}\right.$$

Because the frequency of light remains constant while propagating through a medium, therefore

(6) $$f=\frac{c_{A}}{\lambda_{A}}=\frac{c_{B}}{\lambda_{B}}=\frac{c_{0}}{\lambda_{0}}$$

where $\lambda_{A}$, $\lambda_{B}$, and $\lambda_{0}$ represent the wavelengths of light beams A, B, and in vacuum, respectively.

Furthermore, it is possible to calculate the number of wavelengths experienced by light beams A and B after one rotation around the optical fiber ring, denoted as $k_{A}$ and $k_{B}$, respectively. As follows:

(7) $$\left\{\begin{array}{c} k_{A}=\frac{c_{A}}{\lambda_{A}}t_{A}=\frac{c_{0}}{\lambda_{0}}t_{A}\\ k_{B}=\frac{c_{B}}{\lambda_{B}}t_{B}=\frac{c_{0}}{\lambda_{0}}t_{B} \end{array}\right.$$

Subsequently, when light beams A and B converge, the difference in the number of wavelengths between them is

(8) $$∆k=\left(k_{A}-k_{B}\right)=\left(t_{A}-t_{B}\right)\frac{c_{0}}{\lambda_{0}}=\frac{4\pi R^{2}}{{c_{0}}^{2}-{\left(R\Omega \right)}^{2}}\Omega \frac{c_{0}}{\lambda_{0}}$$

Due to

(9) $$c_{0}\gg R\Omega$$

Equation (8) can be simplified to

(10) $$∆k=\frac{4\pi R^{2}}{{c_{0}}^{2}}\Omega \frac{c_{0}}{\lambda_{0}}=\frac{4\pi R^{2}}{c_{0}\lambda_{0}}\Omega$$

Therefore, the phase difference is

(11) $$∆\Phi =2\pi ∆k=2\pi^{2}\frac{D^{2}}{c_{0}\lambda_{0}}\Omega$$

For an *N*-turn optical fiber, every turn generates a phase difference ∆Φ, and consequently, the total phase difference for an *N*-turn optical fiber ring is

(12) $$∆\Phi =2\pi N∆k=2\pi^{2}N\frac{D^{2}}{c_{0}\lambda_{0}}\Omega$$

## 3. The Thermal Error Model for a Fiber Optic Gyroscope

From a fundamental analysis, derive the theoretical model of thermal errors in optical fibers under conditions of uniform temperature variation. Through extensive experimental regression, obtain the key parameters to provide theoretical support for modeling mass-produced products, addressing overfitting issues arising from modeling with small-scale data, and reducing the dependency on data for subsequent mass-produced product modeling.

### 3.1. The Theoretical Form of the Thermal Error Model

According to Equation (12), when the optical fiber ring is subjected to heating, its diameter changes, leading to a variation in the proportionality factor between the phase difference ∆Φ and the angular velocity Ω (denoted as $K_{∆\Phi /\Omega }$). Assuming the function describing the change in optical fiber ring diameter with temperature is D(∆t), then

(13) $$K_{∆\Phi /\Omega }\left(∆t\right)=2\pi^{2}N\frac{{D(∆t)}^{2}}{c_{0}\lambda_{0}}$$

where the parameters $c_{0},\lambda_{0}$ and *N* are all known values. The key to the model lies in D(∆t). For a circular ring made of a single material, it follows that

(14) $$D\left(∆t\right)=D_{0}+\alpha D_{0}∆t$$

Substituting Equations (13) and (14) into Equation (12), we obtain

(15) $$∆\Phi \left(∆t\right)=\frac{{2N\left({\pi D}_{0}\alpha \right)}^{2}}{c_{0}\lambda_{0}}{∆t}^{2}\Omega +\frac{{4ND_{0}\alpha \left(\pi \right)}^{2}}{c_{0}\lambda_{0}}∆t\Omega +\frac{{2N\left({\pi D}_{0}\right)}^{2}}{c_{0}\lambda_{0}}\Omega$$

where α is the coefficient of thermal expansion, and $D_{0}$ is the diameter of the optical fiber ring when ∆t=0, for example, if taken as a reference at 20 °C, then $D_{0}$ is the diameter at 20 °C. However, Equation (15) is difficult to apply to the actual structure of the optical fiber ring because its interior is composed of multiple materials, making it challenging to determine the comprehensive coefficient of thermal expansion after the composite of various materials.

### 3.2. Method for Determining the Comprehensive Coefficient of Thermal Expansion in the Thermal Error Model

Selecting a specific optical fiber ring, conducting a large-scale experiment, and regressing experimental data to obtain the value of the comprehensive coefficient of thermal expansion, denoted as $\alpha^{*}$. Substituting $\alpha^{*}$ into Equation (15), we can derive

(16) $$∆\Phi \left(∆t\right)=\frac{{2N\left({\pi D}_{0}\alpha^{*}\right)}^{2}}{c_{0}\lambda_{0}}{∆t}^{2}\Omega +\frac{{4ND_{0}\alpha^{*}\left(\pi \right)}^{2}}{c_{0}\lambda_{0}}∆t\Omega +\frac{{2N\left({\pi D}_{0}\right)}^{2}}{c_{0}\lambda_{0}}\Omega$$

Subsequently, we can establish the following model form.

(17) $$y=k_{2}x_{2}+k_{1}x_{1}+k_{0}$$

where,

(18) $$\left\{\begin{array}{c} \begin{array}{c} x_{1}=∆t\\ x_{2}={∆t}^{2} \end{array}\\ y=\frac{∆\Phi }{\Omega } \end{array}\right.$$

According to Equation (18), it is possible to measure the phase difference of the optical fiber ring at different temperatures and different rotational speeds, calculate the measured values of $x_{1},x_{2}$ and y, and then perform regression to obtain the values of $k_{0},k_{1}$, and $k_{2}$. According to Equation (16), it is evident that by utilizing $k_{0},k_{1}$, and $k_{2}$ separately, it is possible to estimate $\alpha^{*}$, as follows

(19) $$\left\{\begin{array}{c} {\alpha^{*}}_{m1}=\sqrt{\frac{k_{2}c_{0}\lambda_{0}}{2N{\left({\pi D}_{0}\right)}^{2}}}\\ {\alpha^{*}}_{m2}=\frac{k_{1}c_{0}\lambda_{0}}{4N{\left(\pi \right)}^{2}D_{0}}\\ {\alpha^{*}}_{m3}=\sqrt{\frac{k_{2}}{k_{0}}} \end{array}\right.$$

To obtain an accurate estimate of α*, it is possible to assess the precision of the three methods separately. First, assess the estimation precision of $k_{0},k_{1}$, and $k_{2}$, as follows

(20) $$\left(\begin{array}{c} \sigma_{k_{0}}\\ \sigma_{k_{1}}\\ \sigma_{k_{2}} \end{array}\right)=\sigma \left(\begin{array}{c} \sqrt{d_{11}}\\ \sqrt{d_{22}}\\ \sqrt{d_{33}} \end{array}\right)$$

where,

(21) diag((ATA)−1)=d11   d22   d33

(22) $$A=\left(\begin{array}{cc} x_{11} & x_{21}\\ \vdots & \vdots \\ x_{1n} & x_{2n} \end{array}\right)$$

(23) $$\sigma =\sqrt{\frac{\sum_{j=1}^{n}{v_{j}}^{2}}{n-2}}$$

(24) $$v_{j}=y_{j}-\hat{y_{j}}=k_{2}x_{2j}+k_{1}x_{1j}+k_{0}$$

where, $x_{i1},\ldots ,x_{in}$ are n measured values of variable $x_{i}$, and $v_{1},\ldots ,v_{n}$ are the estimated residuals when the n measurements are plugged into the model. Ultimately,

(25) $$\alpha^{*}=\frac{\left(\sigma_{{\alpha^{*}}_{m2}}+\sigma_{{\alpha^{*}}_{m3}}\right){\alpha^{*}}_{m1}+\left(\sigma_{{\alpha^{*}}_{m1}}+\sigma_{{\alpha^{*}}_{m3}}\right){\alpha^{*}}_{m2}+\left(\sigma_{{\alpha^{*}}_{m1}}+\sigma_{{\alpha^{*}}_{m2}}\right){\alpha^{*}}_{m3}}{\sigma_{{\alpha^{*}}_{m1}}+\sigma_{{\alpha^{*}}_{m2}}+\sigma_{{\alpha^{*}}_{m3}}}$$

where $\sigma_{{\alpha^{*}}_{m1}},\sigma_{{\alpha^{*}}_{m2}}$ and $\sigma_{{\alpha^{*}}_{m3}}$ represent the estimated standard deviations for the three methods, which can be calculated using the uncertainty synthesis formula based on $\left(\begin{array}{c} \sigma_{k_{0}}\\ \sigma_{k_{1}}\\ \sigma_{k_{2}} \end{array}\right)$ and Equation (19), as shown in Equation (25).

(26) $$\left\{\begin{array}{c} \sigma_{{\alpha^{*}}_{m1}}=\sqrt{{\left(\frac{\partial {\alpha^{*}}_{m1}}{\partial k_{2}}\sigma_{k_{2}}\right)}^{2}+{\left(\frac{\partial {\alpha^{*}}_{m1}}{\partial D_{0}}\sigma_{D_{0}}\right)}^{2}}\\ \sigma_{{\alpha^{*}}_{m2}}=\sqrt{{\left(\frac{\partial {\alpha^{*}}_{m2}}{\partial k_{1}}\sigma_{k_{1}}\right)}^{2}+{\left(\frac{\partial {\alpha^{*}}_{m2}}{\partial D_{0}}\sigma_{D_{0}}\right)}^{2}}\\ \sigma_{{\alpha^{*}}_{m3}}=\sqrt{{\left(\frac{\partial {\alpha^{*}}_{m3}}{\partial k_{0}}\sigma_{k_{0}}\right)}^{2}+{\left(\frac{\partial {\alpha^{*}}_{m3}}{\partial k_{2}}\sigma_{k_{2}}\right)}^{2}} \end{array}\right.$$

where, $\sigma_{D_{0}}$ represents the standard deviation of the optical fiber ring diameter measurements.

### 3.3. Method for Establishing the Thermal Error Model

For subsequent mass-produced optical fiber rings, the thermal error model has the form of Equations (16) and (17). After obtaining the value of $\alpha^{*}$, even if its number of turns and inner diameter change, as long as the internal material remains unchanged, it is possible to calculate the theoretical values of $k_{0},k_{1}$, and $k_{2}$ in the corresponding thermal error model of the optical fiber ring. However, considering the process differences, each optical fiber ring still needs to undergo thermal error testing experiments to establish a high-precision thermal error model based on data.

However, during the model-building process, it is possible to effectively integrate the theoretical values of $k_{0},k_{1}$, and $k_{2}$ with the testing data, reducing the dependency of model construction on the quantity of testing data samples. The specific method involves adding the theoretical values of $k_{0},k_{1}$, and $k_{2}$ as regularization terms to the objective function of the regression algorithm when performing regression on testing data, as shown in Equation (27).

(27) $$\underset{k_{0},k_{1},k_{2}}{\min}\Gamma =((1-\eta )\sum_{j=1}^{n}{v_{j}}^{2}+\eta \sum_{i=0}^{2}{\left({k_{i}}^{\;}-{k_{i}}^{*}\right)}^{2})$$

where ${k_{i}}^{*}$ represents the theoretical value of $k_{i}$, and *η* = [0, 1] is the weight of the regularization term. This algorithm is actually an extension of ridge regression. If ${k_{i}}^{*}$ is set to 0, Equation (27) becomes ridge regression. Therefore, with a small weight for the regularization term, it can significantly reduce the estimation error of model coefficients. In this study, *η* = 0.1 was chosen. The solution method is as shown in Equation (28).

(28) $$\left\{\begin{array}{c} \frac{\partial \Gamma }{\partial k_{0}}=0\\ \vdots \\ \frac{\partial \Gamma }{\partial k_{2}}=0 \end{array}\right.\Rightarrow \left(\begin{array}{c} k_{0}\\ k_{1}\\ k_{2} \end{array}\right)={\left((1-\eta )A^{T}A+\eta I\right)}^{-1}{((1-\eta )A}^{T}Y+\eta K^{*})$$

where *I* is the identity matrix, $Y={\left(y_{1},\ldots ,y_{n}\right)}^{T}$ represents the testing data for variable y, and $K^{*}={\left({k_{0}}^{*},{k_{1}}^{*},{k_{2}}^{*}\right)}^{T}$.

## 4. Verification Experiment

To validate the effectiveness of the fiber optic gyroscope’s thermal error model, temperature variation experiments were conducted on the fiber optic gyroscope for detection.

### 4.1. Experimental Plan

**Figure 3:**
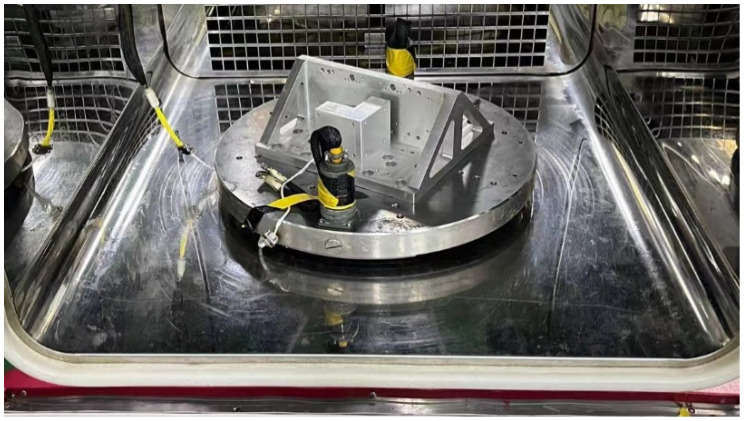
Experimental setup for thermal deformation error testing of fiber optic gyroscope.

Place the fiber optic gyroscope with dimensions of 50 × 50 in a temperature chamber, and the optical fiber phase variation was measured within the temperature range of −18 to 65 °C and the angular velocity range of −860 to 860°/h. The experimental setup, as shown in Figure 3 and Table 1, involved mounting the gyroscope under test inside a temperature-controlled chamber with a rotating platform. The temperature was gradually reduced to −18 °C at a rate of 2 °C/min, followed by a 2 h insulation period. The rotating platform was then tested at different speeds: ±860, ±500, ±360, ±180, ±90, ±30, ±10, ±1, ±0.5, ±0.3, and ±0.1°/h, and so on, at 0 °C, 25 °C, 40 °C, and 65 °C, replicating the same tests at each temperature point.

### 4.2. Experimental Data

**Figure 4:**
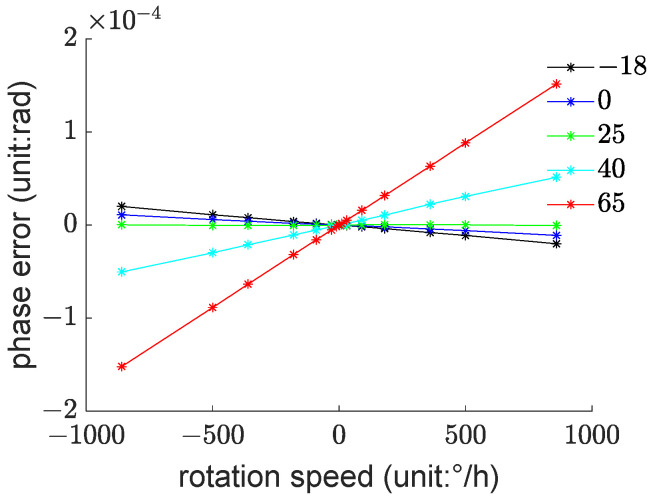
Experimental Data for Fiber Optic Gyroscope Thermal Deformation Error Testing.

As shown in Figure 4, these are the phase errors of the fiber optic gyroscope at each temperature test point.

For better visualization, the raw data in this study have been processed. Figure 4 represents the difference between the phase measurement values at each test point and the theoretical values under ideal conditions at 20 °C. These differences are referred to as phase errors. The theoretical values are calculated by substituting the angular velocity values into Equation (12).

It can be observed that as the temperature gradually increases, the phase error exhibits clear non-linearity but maintains a monotonous changing trend. The slope of the phase error with respect to angular velocity changes from negative values to positive values gradually, and with the rise in temperature, the rate of increase also becomes greater.

### 4.3. Experimental Results

To validate the effectiveness of the thermal error modeling, this study chose to build the model using data from three temperature test points: 0 °C, 25 °C, and 40 °C. Subsequently, the data from two temperature test points, −18 °C and 65 °C, were used for prediction, serving as a test of the model’s predictive accuracy for data not included in the modeling process.

First, based on Equation (25), the comprehensive coefficient of thermal expansion for the optical fiber ring was estimated to be $0.76\times 10^{-5}$. This value was then used in Equation (16) to calculate the model’s theoretical values. Subsequently, the theoretical values and measurement data were jointly used in Equation (28) to establish the model, referred to as the Theoretical-Data-Driven model or TD-model. For the purpose of comparison, this study directly performed regression on the measurement data to establish a control model known as the Pure Data model or OD-model.

**Figure 5:**
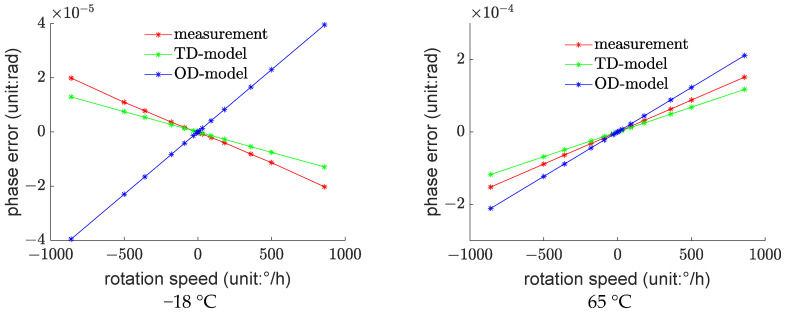
Comparison of Predictive Performance of the Fiber Optic Gyroscope Thermal Deformation Error Model.

Subsequently, based on the original measurement data, the input values required for the model predictions, namely, ${∆t}^{2}\Omega$, ∆tΩ, and Ω, as in Equation (16), were computed. These values were separately used in the TD-model and OD-model to obtain model predictions, which were then compared with the actual measurement values, as shown in Figure 5.

It can be observed that the TD-model significantly outperforms the OD-model, especially in the −18 °C environment where the OD-model exhibits predictive results that are completely opposite to the measurement values.

To quantitatively assess the predictive performance of the two modeling approaches, the relative root mean square error (RMSE) for each model’s predictions of the measurement data was calculated. The RMSE calculation method is shown in Equation (29).

(29) $$RMSE=\sqrt{\frac{\sum_{k=1}^{n}{\left(y_{k}^{\;}-{\hat{y}}_{k}^{\;}\right)}^{2}}{n}}$$

where ${\hat{y}}_{k}$ represents the model’s prediction, and ${\hat{y}}_{k}$ represents the measured result. According to Equation (11), *RMSE* reflects the sum of squares of differences between the model’s predicted results and the actual measurement results. The larger the differences, the larger the *RMSE*. Using the range of measured values as the range, the relative *RMSE* as a percentage of the full-scale range was calculated, as shown in Table 2.

The data indicate that the predictive accuracy of the TD-model is improved by 58% compared to the OD-model, demonstrating the effectiveness of the proposed algorithm.

## 5. Discussion

Discussing and analyzing the experimental results.

One of the most significant controversies encountered during the research process is why the predictive results of the OD-model at −18 °C are opposite to the actual measurement values, while the TD-model provides accurate results.

**Figure 6:**
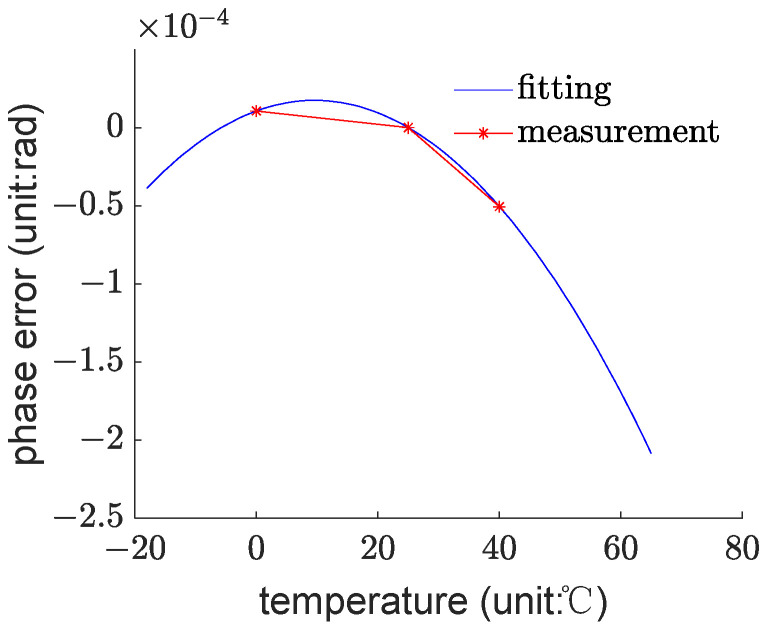
Observation of Measurement Data and Fitting for Phase Error vs. Temperature (−860°/h, 0 °C, 25 °C, 40 °C).

The modeling data used for this study come from measurements taken at temperatures of 0 °C, 25 °C, and 40 °C. As observed in Figure 4, examining the variation in phase errors at these three temperature points reveals that if we fix the gyroscope at a certain rotational speed, the phase error decreases non-linearly and monotonically with respect to temperature. For example, at a rotational speed of −860°/h, plotting the variation curve of phase error with temperature and fitting it with the quadratic model results in the pattern shown in Figure 6.

It can be observed that although the measurement data exhibit a monotonically decreasing trend, the fitting results suggest that the most appropriate quadratic curve assumes that the phase error is monotonically increasing with temperature near 0 °C, thereby leading to an overfitting issue. For other angular velocities, the same conclusion can be drawn, and thus, further elaboration is not necessary.

This issue is one of the reasons for introducing the TD-model. Fiber optic gyroscope testing experiments are relatively complex, making it difficult to conduct a large number of experiments, resulting in limited data. These limited data can easily lead to overfitting. The TD-model introduces a theoretical model to correct the distortion caused by overfitting. By observing Equation (28), it can be seen that the TD-model’s calculation formula is actually a variant of ridge regression [21]. If $K^{*}$ is set to 0, it becomes ridge regression. According to the properties of ridge regression [22], a slight increase in the value of η, although causing a slight deviation of the model’s expectation from the true value, leading from an unbiased regression to a biased regression, can effectively suppress overfitting. This significantly increases the probability of the model falling close to the true value. One difference between the TD-model and ridge regression is that if ridge regression increases the value of η, the model coefficients tend to approach zero, whereas the TD-model makes the model coefficients tend to be closer to the theoretical values of the true values. Thus, while suppressing overfitting, the TD-model significantly reduces the degree to which the model’s expectation deviates from the true value, allowing for the establishment of a more effective model.

Furthermore, this paper analyzes the stability of sensitivity and zero drift of the fiber optic gyroscope after compensation. Sensitivity and zero drift are calculated using the phase signals before and after compensation, respectively. The signal before compensation is the original phase measurement value, and the signal after compensation is the original phase measurement value minus the phase error predicted by the TD-model.

For the fiber optic gyroscope, sensitivity is defined in this paper as the phase change caused by unit rotational speed variation, as shown in Equation (30), where the output is the phase signal of the fiber optic gyroscope, and the input is the rotational speed. The specific calculation method involves the least squares fitting coefficients between the output and input at each temperature point.

(30) $$sensitivity=\frac{∆\Phi }{\Omega }$$

Zero drift refers to the phase error when the rotational speed is 0°/h, and in this paper, 0.1°/h phase error is taken as the zero drift.

**Figure 7:**
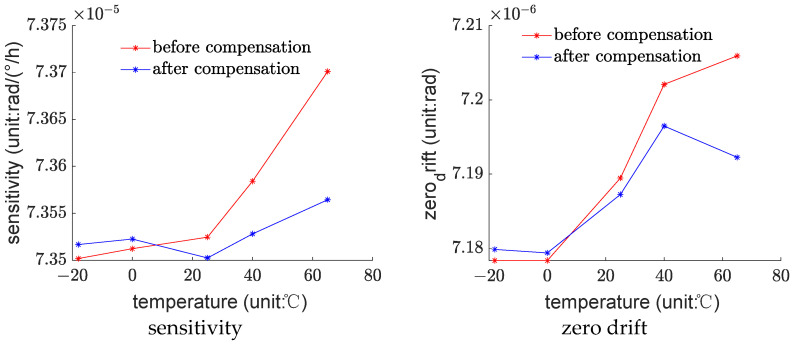
Relationship between sensitivity, zero drift, and temperature.

As shown in Figure 7, the data illustrate the variations in sensitivity and zero drift with temperature before and after compensation.

As shown in Figure 7, after compensation, the temperature stability of sensitivity has significantly improved, but the improvement in zero drift is not apparent. According to Equations (12) and (30), it can be determined that the theoretical value of sensitivity is

(31) $$2\pi^{2}N\frac{D^{2}}{c_{0}\lambda_{0}}$$

Furthermore, theoretically, sensitivity will be influenced by the deformation of the fiber structure. However, the theoretical value of zero drift, obtained by setting Ω = 0 and substituting into Equation (12), is a constant 0, indicating that it theoretically will not be affected by structural changes. Therefore, compared to zero drift, sensitivity is more susceptible to the influence of temperature, and after compensation, stability can also be improved.

## 6. Conclusions

This paper has conducted modeling and analysis of the thermal deformation error in fiber optic gyroscopes. By combining theoretical derivation with experimental data, a new thermal error modeling algorithm has been proposed, significantly improving the modeling accuracy of thermal deformation error in fiber optic gyroscopes. The specifics are as follows.

Based on the working principles of fiber optic gyroscopes, a theoretical model was derived for the phase error induced by thermal deformation in the fiber optic ring and its relationship with temperature and angular velocity. The model reveals that the phase error in fiber optic gyroscopes is quadratically related to temperature. However, obtaining a quantitative theoretical model relies on accurate thermal expansion coefficients of the materials used in the fiber optic ring. Since the fiber optic ring is composed of multiple materials in combination, determining the thermal expansion coefficients theoretically becomes challenging.

Thermal deformation error measurement experiments were conducted on a specific model of a fiber optic ring. Phase errors relative to the ideal environment at 20 °C were measured at different angular velocities and temperatures. The experimental results revealed a nonlinear correlation between phase error and temperature, aligning with theoretical expectations. However, due to the complexity of the experiments and the difficulty in conducting them on a large scale, direct modeling using experimental data resulted in overfitting issues and poor predictive accuracy of the model. This paper introduces a novel modeling algorithm that combines the theoretically derived model with experimental data, effectively mitigating the overfitting problem. The model established is referred to as the Theoretical-Data-Driven model, abbreviated as TD-model. It has been validated that the TD-model significantly enhances the predictive accuracy of phase errors induced by thermal deformation in fiber optic gyroscopes. Compared to a model based solely on experimental data, the TD-model demonstrates a 58% improvement in predictive accuracy, thus confirming the model’s effectiveness.

## Figures and Tables

**Table 1:** Experimental parameters for thermal deformation error testing of fiber optic gyroscope.

Temperature Test Points (°C)	Angular Velocity Test Points (°/h)		Fiber Optic Ring Parameters			
−18, 0, 25, 40, 65	±860, ±500, ±360, ±180, ±90, ±30, ±10, ±1, ±0.5, ±0.3, ±0.1	Diameter (mm)	Winding method	Number of layers	Fiber optic length (m)	Wavelength (nm)
		45.6	Fourfold symmetry	20	330	650 nm

**Table 2:** Relative *RMSE* for Predictions of Fiber Optic Gyroscope Thermal Deformation Error Model.

	TD-Model	OD-Model
*RMSE*	3.0716%	7.3777%

## Data Availability

The data underlying the results presented in this paper are not publicly available at this time but may be obtained from the authors upon reasonable request.
